# Critical role of TNF-alpha-TNFR1 signaling in intracranial aneurysm formation

**DOI:** 10.1186/2051-5960-2-34

**Published:** 2014-03-31

**Authors:** Tomohiro Aoki, Miyuki Fukuda, Masaki Nishimura, Kazuhiko Nozaki, Shuh Narumiya

**Affiliations:** 1 Innovation Center for Immunoregulation Technologies and Drugs (AK project), Kyoto University Graduate School of Medicine, Konoe-cho Yoshida, Sakyo-ku, Kyoto City, Kyoto 606-8501, Japan; 2 Core Research for Evolutional Science and Technology (CREST), Medical Innovation Center, Kyoto University Graduate School of Medicine, 53 Kawahara-cho Shogoin, Sakyo-ku, Kyoto City, Kyoto 606-8507, Japan; 3 Department of Neurosurgery, Kyoto University Graduate School of Medicine, 54 Kawahara-cho Shogoin, Sakyo-ku, Kyoto City, Kyoto 606-8507, Japan; 4 Department of Neurosurgery, Shiga University of Medical Science, Tsukinowa-cho Seta, Otsu City, Shiga 520-2192, Japan

**Keywords:** Inflammation, Intracranial aneurysm, Macrophage, MCP-1, NF-kappaB, TNF-alpha

## Abstract

**Background:**

Intracranial aneurysm (IA) is a socially important disease due to its high incidence in the general public and the severity of resultant subarachnoid hemorrhage that follows rupture. Despite the social importance of IA as a cause of subarachnoid hemorrhage, there is no medical treatment to prevent rupture, except for surgical procedures, because the mechanisms regulating IA formation are poorly understood. Therefore, these mechanisms should be elucidated to identify a therapeutic target for IA treatment. In human IAs, the presence of inflammatory responses, such as an increase of tumor necrosis factor (TNF)-alpha, have been observed, suggesting a role for inflammation in IA formation. Recent investigations using rodent models of IAs have revealed the crucial role of inflammatory responses in IA formation, supporting the results of human studies. Thus, we identified nuclear factor (NF)-kappaB as a critical mediator of inflammation regulating IA formation, by inducing downstream pro-inflammatory genes such as MCP-1, a chemoattractant for macrophages, and COX-2. In this study, we focused on TNF-alpha signaling as a potential cascade that regulates NF-kappaB-mediated IA formation.

**Results:**

We first confirmed an increase in TNF-alpha content in IA walls during IA formation, as expected based on human studies. Consistently, the activity of TNF-alpha converting enzyme (TACE), an enzyme responsible for TNF-alpha release, was induced in the arterial walls after aneurysm induction in a rat model. Next, we subjected tumor necrosis factor receptor superfamily member 1a (TNFR1)-deficient mice to the IA model to clarify the contribution of TNF-alpha-TNFR1 signaling to pathogenesis, and confirmed significant suppression of IA formation in TNFR1-deficient mice. Furthermore, in the IA walls of TNFR1-deficient mice, inflammatory responses, including NF-kappaB activation, subsequent expression of MCP-1 and COX-2, and infiltration of macrophages into the IA lesion, were greatly suppressed compared with those in wild-type mice.

**Conclusions:**

In this study, using rodent models of IAs, we clarified the crucial role of TNF-alpha-TNFR1 signaling in the pathogenesis of IAs by inducing inflammatory responses, and propose this signaling as a potential therapeutic target for IA treatment.

## Introduction

Intracranial aneurysm (IA) is a lesion with a regional bulging of intracranial arteries, usually located at bifurcation sites. IA is a common disease in the general public, with a prevalence of 1–5 percent [1], and is a major cause of subarachnoid hemorrhage [2]. Subarachnoid hemorrhage continues to be responsible for high mortality and morbidity rates, despite advances in medical technologies [3], and therefore, the prevention of pre-existing IA rupture is imperative. Furthermore, IAs have a remarkable negative impact on society, not only due to death or complications resulting from subarachnoid hemorrhage, but also due to the anxiety associated with potential rupture. Indeed, a recent report demonstrated that the quality of life of patients with IAs was significantly limited [4] even if IAs did not rupture. Importantly, treatment of IAs was also shown to restore the social activity of these patients. In this sense, the treatment of IAs is socially important. However, to date, except for surgical procedures, there is no available medical treatment to prevent the rupture of IAs. Given the severity and negative social impact of a resulting subarachnoid hemorrhage after rupture, the mechanisms underlying IA formation and rupture should be investigated to develop a novel medical treatment for IAs.

Various studies using human IA specimens have revealed the involvement of active inflammatory responses in IA lesions, such as the expression of inflammatory cytokines, the infiltration of inflammatory cells into lesions, and the positive linkage of inflammatory-gene polymorphisms with IAs [5-9]. Furthermore, recent experiments using animal models of IAs support the interpretation of human studies that inflammatory responses in intracranial arterial walls regulate IA formation and progression. Among factors regulating inflammatory processes, we have revealed a critical role for nuclear factor (NF)-kappaB in the pathogenesis of IA formation. NF-kappaB is activated in endothelial cells and macrophages in IA walls during IA formation, and NF-kappaB p50 subunit deficiency or inhibition by decoy oligonucleotides or compounds with anti-NF-kappaB effects significantly suppresses IA formation and progression in rodent models [10,11]. In addition, the inhibition of NF-kappaB activation in IA walls suppresses the expression and production of downstream pro-inflammatory factors regulating IA formation, such as matrix metalloproteinase-9 (MMP-9), monocyte chemoattractant protein-1 (MCP-1), and interleukin-1 beta (IL-1beta) [10,11]. However, because NF-kappaB mediates many physiological processes, it is not a suitable therapeutic target. Indeed, despite the crucial role of NF-kappaB in various inflammatory diseases, no anti-NF-kappaB therapy has been established.

Tumor necrosis factor (TNF)-alpha is a cytokine that can strongly activate NF-kappaB, and TNF-alpha signaling has been implicated in the pathogenesis of human IAs. For example, increased TNF-alpha mRNA expression in lesions as determined by reverse transcription PCR (RT-PCR) [6], increased plasma TNF-alpha concentration in patients with IAs [12], and the possible linkage of TNF-alpha signaling with IA rupture in whole-genome expression profile analysis [9], have been reported. Furthermore, a positive correlation between single nucleotide polymorphisms in the TNF-alpha gene and increased risk of incidence and rupture, has been identified [13,14]. Based on these findings, in this study, we examined the contribution of TNF-alpha signaling to IA formation using tumor necrosis factor receptor superfamily member 1a (TNFR1)-deficient mice.

## Materials and methods

### Rodent IA models and histological analysis of induced IA

All of the following experiments, including animal care and use, complied with the National Institute of Health’s Guide for the Care and Use of Laboratory Animals and were approved by the Institutional Animal Care and Use Committee of Kyoto University Graduate School of Medicine.

Male Sprague–Dawley rats were purchased from Japan SLC (Shizuoka, Japan). TNFR1-deficient mice were purchased from The Jackson Laboratory (Bar Harbor, ME, USA). Animals were maintained on a light/dark cycle of 14 h/10 h, and had free access to chow and water. To induce IA, under general anesthesia by intraperitoneal injection of pentobarbital sodium (50 mg/kg), 7-week-old male rats or mice were subjected to ligation of the left carotid artery and systemic hypertension, induced by the combination of salt overloading and ligation of the left renal artery. This procedure is designed to increase hemodynamic stress, the trigger of IA formation [15-17], on the bifurcation site of intracranial arteries [18-20]. Immediately after the surgical procedure, animals were fed a special chow containing 8% sodium chloride and 0.12% 3-aminopropionitrile (Tokyo Chemical Industry, Tokyo, Japan), an inhibitor of lysyl oxidase that catalyzes the cross-linking of collagen and elastin. IA induction at the right anterior cerebral artery (ACA) and olfactory artery (OA) bifurcation, the contralateral side of the carotid ligation was assessed at times indicated in the corresponding Figure legends [18-20]. In histological analyses, after measurement of systemic blood pressure by the tail-cuff method, animals were deeply anesthetized by intraperitoneal injection with a lethal dose of pentobarbital sodium, and transcardially perfused with 4% paraformaldehyde. The right ACA-OA bifurcation including the IA lesion was then stripped, and serial frozen sections were made. The IA lesion was defined by the disruption of the internal elastic lamina, as visualized by Elastica van Gieson staining.

### Immunohistochemistry

**Figure 1:**
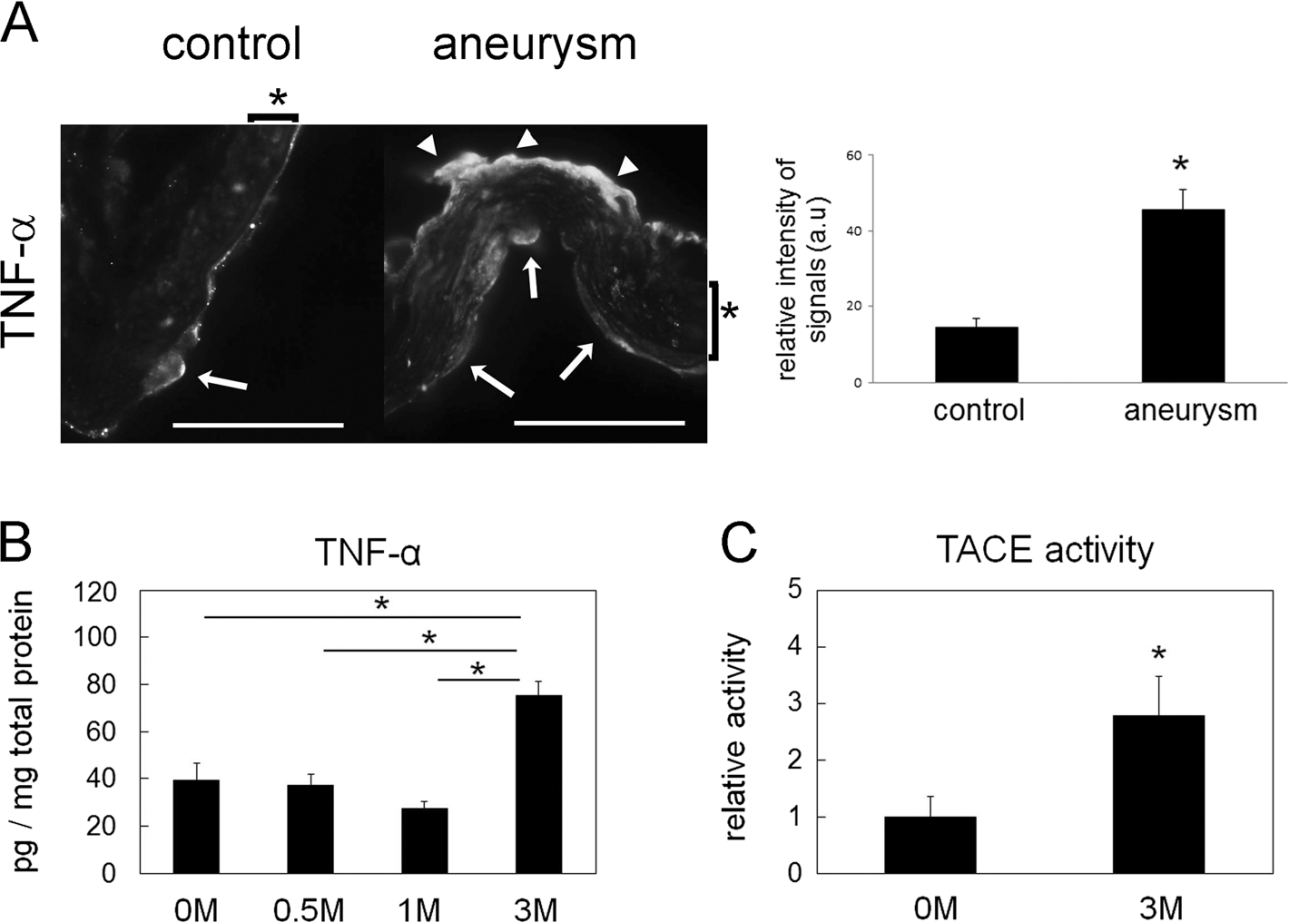
**Increased TNF-alpha content in the IA lesion. (A)** Immunohistochemistry to detect TNF-alpha in IA walls located at right anterior cerebral artery-olfactory artery bifurcations in a rat model. Immunohistochemistry to detect TNF-alpha in control arterial and IA walls from rats, 3 months after aneurysm induction, is shown. The white arrow and arrow head indicate positive staining in endothelial cells and outer membranes, respectively. The star in the left panel indicates the media of arterial walls. Bar = 50 um. In the right panel, the relative intensities of positive signals in IA walls from the left panel are shown. n = 3 in each group, and *indicates p < 0.05. All bars indicate the mean ± SEM. **(B)** Increased TNF-alpha content in intracranial arteries after aneurysm induction. TNF-alpha content was analyzed by multi-suspension array using intracranial arteries from rats at indicated time points after aneurysm induction. n = 6 in each group. All bars indicate the mean ± SEM, and *indicates p < 0.05. M; month after aneurysm induction. **(C)** Enhanced tumor necrosis factor alpha converting enzyme (TACE) activity in intracranial arteries after aneurysm induction. TACE activity was measured as explained in the Methods section. n = 4 in both groups. All bars indicate the mean ± SEM, and *indicates p < 0.05. M; month after aneurysm induction.

**Figure 2:**
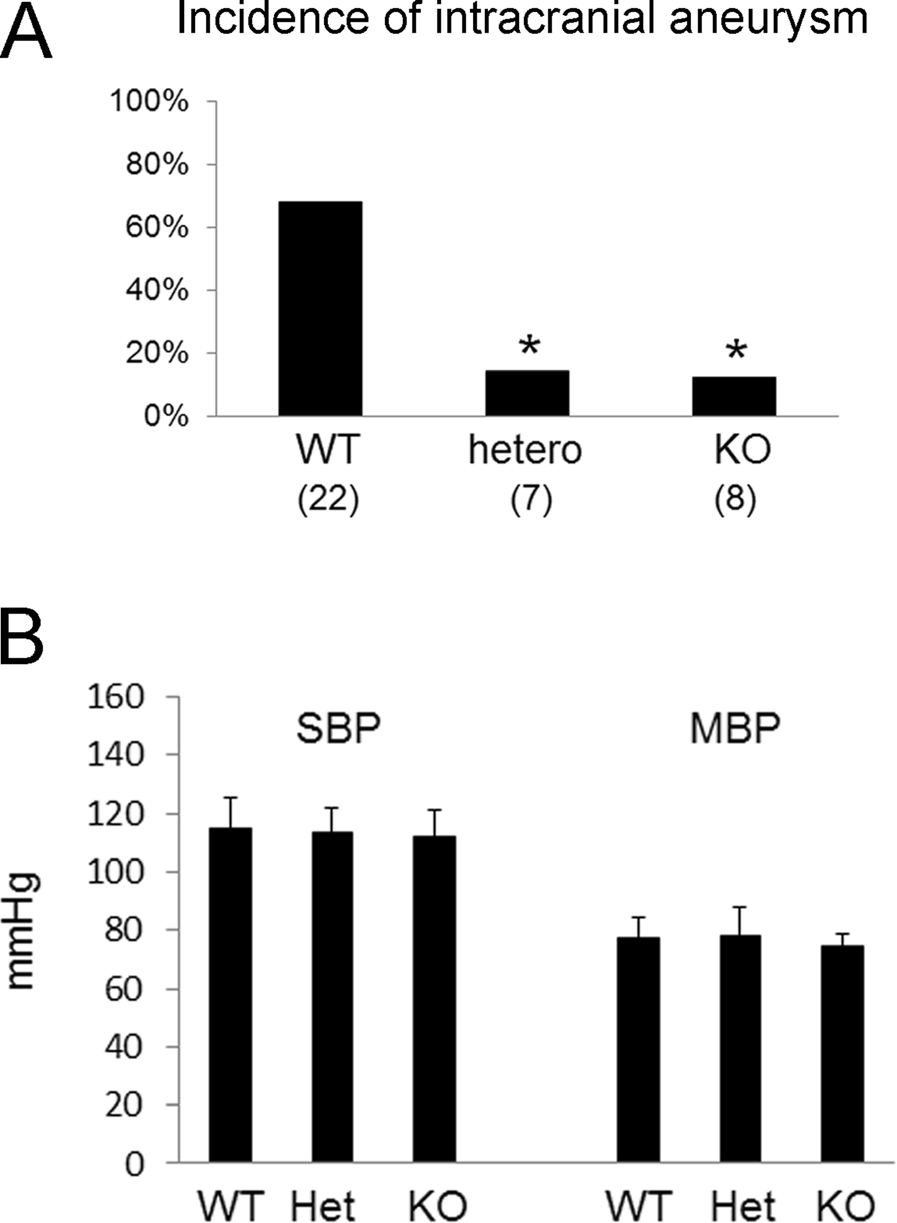
**Suppression of IA formation in TNFR1-deficient mice. (A)** Incidence of IAs in wild-type (WT), TNFR1-heterozygous (hetero), and TNFR1-deficient (KO) mice. IA was defined as a lesion with disrupted internal elastic lamina by Elastica van Gieson staining. The number of mice used for each genotype is shown in parentheses. *indicates p < 0.05. **(B)** Systemic blood pressure in each genotype (WT, wild-type mouse; Het, TNFR1-heterozygous mouse; KO, TNFR1-deficient mouse) after aneurysm induction. Systemic blood pressure (SBP, systolic blood pressure; MBP, mean blood pressure) was measured by the tail-cuff method. All bars indicate the mean ± SEM.

Immunohistochemical analyses were performed as previously described [19]. Briefly, at the indicated period after aneurysm induction, 5-um-thick frozen sections were made as described above. After blocking with 3% donkey or goat serum (Jackson ImmunoResearch, Baltimore, MD, USA), slices were incubated with primary antibodies followed by incubation with fluorescence-labeled secondary antibodies (Jackson ImmunoResearch). Finally, fluorescent images were acquired through a confocal fluorescence microscope system (CTR6500, Leica Microsystems, Tokyo, Japan). Representative images from at least 3 independent samples are shown in Figures 1 and 2. The relative intensity of positive staining in IA walls from each experiment was measured by imaging software and statistically analyzed.

The following primary antibodies were used: mouse monoclonal anti-smooth muscle alpha actin antibody (Thermo scientific, Waltham, MA, USA), rabbit monoclonal anti-phospho NF-kappaB p65 (ser536) antibody (Cell Signaling Technology, Danvers, MA, USA), rat monoclonal anti-TNF-alpha antibody (Lifespan Bioscience, Seattle, WA, USA), goat polyclonal anti-MCP-1 antibody (Santa Cruz Biotechnology, Dallas, TX, USA), rabbit polyclonal anti-COX-2 antibody (Cayman Chemicals, Ann Arbor, MI, USA), rat monoclonal anti-F4/80 antibody (Abcam, Cambridge, UK).

### Macrophage count

Macrophages were defined as cells positive for F4/80 staining in immunohistochemistry. The number of infiltrated macrophages was calculated within 10000 um^2^ around the dome of induced aneurysms (wild-type; n = 7, TNFR1-deficient mouse; n = 5).

### Quantitative real-time PCR analysis

RNA purification from the ring of Willis of mice 5 months after aneurysm induction (n = 4 in wild-type and 3 in TNFR1-deficient mice) and subsequent reverse transcription were performed using a RNeasy Fibrous Tissue Mini Kit (QIAGEN, Hilden, Germany) and a High Capacity cDNA Reverse Transcription Kit (Life Technologies Corporation, Carlsbad, CA, USA), respectively, according to the manufacturers’ instructions. For the quantification of gene expression, PCR reactions were performed using the LightCycler 480 system with a SYBR Premix Ex Taq II (Takara Bio Inc., Shiga, Japan), using the expression of beta-actin as an internal control.

The following primers (forward and reverse, respectively) were used in experiments: 5′-tctgcagccatttccttctctcc-3′ and 5′-aaaggcctccattgaccagagc-3′ for COX-2, 5′-atgtctggacccattccttcttgg-3′ and 5′-tcccaatgagtaggctggagagc-3′ for MCP-1, 5′-gctcgttgccaatagtgatgacc-3′ and 5′-cgtgcgtgacatcaaagagaagc-3′ for beta-actin.

### Quantification of TNF-alpha protein content

The TNF-alpha protein content of intracranial arteries with aneurysm induction was examined using multiplex suspension array techniques, according to the manufacturer’s instructions. Briefly, intracranial arteries were collected from 6 rats at each indicated time period after aneurysm induction, and total protein was purified by Bio-Plex Cell Lysis Kit (Bio-Rad, Hercules, CA, USA). TNF-alpha protein content was then quantified by Bio-Rad DC Protein Assay (Bio-Rad), using the Luminex 2000 System (Luminex, Tokyo, Japan) for detection, and DNASIS Plex version 2.5 (HitachiSoft, Tokyo, Japan) for analysis, according to the manufacturer’s instructions.

### Measurement of TNF-alpha converting enzyme (TACE) activity

The total protein from intracranial arteries after aneurysm induction was purified by Complete M Lysis kit (Roche, Basel, Switzerland) according to the manufacturer’s instructions (n = 4 in each group). Each experiment used 25 ug of protein. Total protein and the fluorescence-labeled synthetic substrate for TACE (5-FAM-Ser-Pro-Leu-Ala-Gln-Ala-Val-Arg-Ser-Ser-Ser-Arg-Lys; BioMol, Plymouth, PA, USA) were incubated in buffer containing 50 mM Tris–HCl (pH 7.4), 25 mM NaCl and 4% glycerol for 1 h at 37°C. Enzyme activity was evaluated based on the increase in fluorescence at 535 nm (excitation 485 nm).

### Statistical analysis

Data are shown as the mean ± SEM, and 2 groups were statistically compared using the Mann–Whitney U test. Statistical comparisons among more than 2 groups were conducted using the Kruskal − Wallis test followed by the Steel − Dwass test. The incidence of IAs was analyzed by Fisher’s exact test. A p value smaller than 0.05 was defined as statistically significant.

## Results

### Increased TNF-alpha content in IA lesion in a rat model

In this study, we used the rodent model of IAs to examine the contribution of TNF-alpha signaling to IA formation. In this model, IA was induced through increased hemodynamic stress, a trigger of IAs in humans [15-17], loaded on the bifurcation sites of intracranial arteries. Three months after aneurysm induction, IA, defined as a lesion with disrupted internal elastic lamina, was induced at the right ACA-OA bifurcation in all rats.

The immunoreactivity of TNF-alpha was remarkably increased in IA lesions compared with control intracranial arteries, especially in endothelial cells and the outer membrane, and the difference in relative intensity of positive signals in IA walls was statistically significant (n = 3 in each group, p = 0.049) (Figure 1A). Consistent with these results, the content of TNF-alpha in the intracranial artery, examined by multi-suspension array, was significantly increased in rats with advanced stage IAs three months after aneurysm induction (0 months, 39.4 ± 7.2 pg/mg total protein; 0.5 months, 37.0 ± 4.8 pg/mg total protein; 1 month, 27.3 ± 3.0 pg/mg total protein; 3 months, 75.2 ± 6.1 pg/mg total protein; n = 6 in each group, 0 months compared to 3 months, p = 0.0497; 0.5 months compared to 3 months, p = 0.0497; 1 month compared to 3 months, p = 0.020) (Figure 1B). TNF-alpha is released from the cytoplasmic membrane through shedding, by the action of TACE. We therefore examined the activity of TACE in intracranial arteries including IA lesions, from a rat model using labeled synthetic substrate. As a result, in intracranial arteries from rats with aneurysm induction, the activity of TACE was significantly increased compared to control rats, suggesting up-regulation of TNF-alpha production in IA lesions, consistent with increased TNF-alpha content in IA lesions (0 months, 1.00 ± 0.36-fold, n = 4; 3 months, 2.78 ± 0.71-fold, n = 4; 0 months compared to 3 months, p = 0.028) (Figure 1C).

### Suppression of IA formation due to TNFR1 deficiency

Next, to clarify the involvement of TNF-alpha signaling in IA formation, we used mice deficient in its receptor, TNFR1, based on the finding that TNFR1 was abundantly expressed in intracranial arteries (data not shown). As a result, 15 out of 22 wild-type mice, 1 out of 7 TNFR1-heterozygous, and 1 out of 8 TNFR1-deficient mice, had IAs at right ACA-OA bifurcations. Statistically, the incidence of IA was significantly suppressed in TNFR1-heterozygous and TNFR1-deficient mice compared with wild-type mice (wild-type compared to TNFR1-heterozygous, p = 0.026, wild-type compared to TNFR1-deficient, p = 0.012) (Figure 2A), suggesting a critical role for TNF-alpha-TNFR1 signaling in IA formation. Because IA formation in the mouse model was greatly influenced by systemic blood pressure, we confirmed the independence of genotypes from systemic blood pressure after aneurysm induction (systolic blood pressure: wild-type mice, 115.0 ± 10.3 mmHg, n = 8; TNFR1-heterozygous mice, 113.0 ± 9.0 mmHg, n = 7; TNFR1-deficient mice, 112.3 ± 8.6 mmHg, n = 8; mean blood pressure: wild-type mice, 77.3 ± 7.2 mmHg, n = 8; TNFR1-heterozygous mice, 78.1 ± 9.8 mmHg, n = 7; TNFR1-deficient mice, 74.8 ± 3.9 mmHg, n = 8) (Figure 2B).

### Suppression of inflammatory responses due to TNF-alpha-TNFR1 signaling deficiency

Previously, we demonstrated the crucial role of inflammatory responses in IA formation [10,11,19,21,22]. In this process, NF-kappaB activation and subsequent enhancement of macrophage infiltration into intracranial arteries via NF-kappaB mediated-induction of MCP-1, a major chemoattractant of macrophages in affected sites, are essential for IA formation [10,11,19,21,22]. We therefore analyzed whether TNF-alpha-TNFR1 signaling deficiency would attenuate this process.

**Figure 3:**
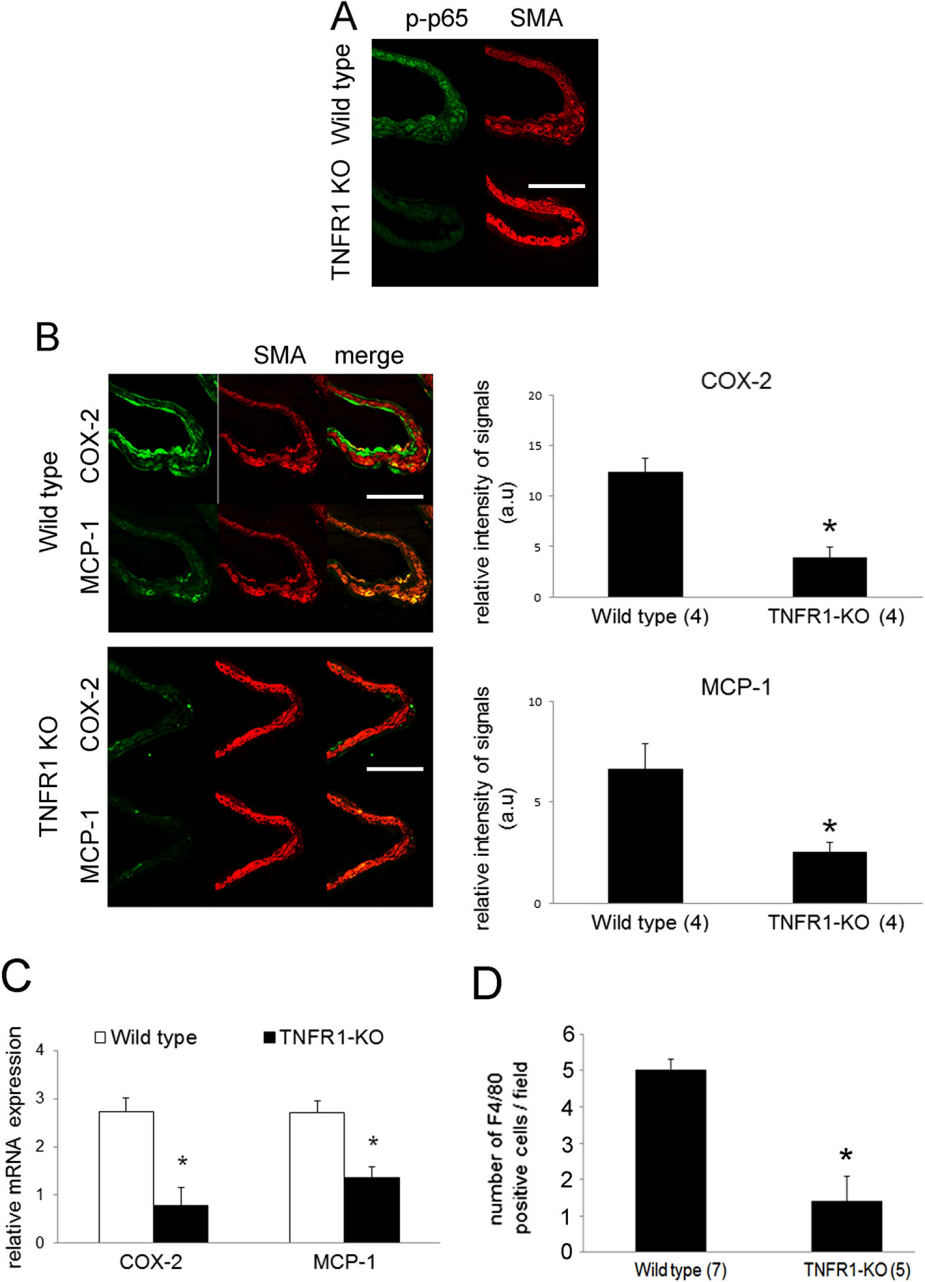
**Suppression of inflammatory responses in IA walls resulting from defective TNFR1 signaling. (A)** Immunohistochemistry to detect the phosphorylated form of the NF-kappaB p65 subunit (p-p65) in IA walls located at right anterior cerebral artery-olfactory artery bifurcations in wild-type and TNFR1-deficient (TNFR1 KO) mice. Immunohistochemistry for anti- smooth muscle alpha actin (SMA) was used to indicate the media of IA walls. Bar = 50 um. **(B)** Immunohistochemistry for COX-2 (upper columns), or MCP-1 (lower columns), and SMA (red color) in IA walls of wild-type and TNFR1 KO mice. Bar = 50 um. In the right panels, the relative intensities of positive signals of COX-2 and MCP-1 in IA walls from the left panels are shown. The number of mice used is shown in parentheses. *indicates p < 0.05. All bars indicate the mean ± SEM. **(C)** mRNA expression of COX-2 and MCP-1 in wild-type and TNFR1 KO mice from quantitative real-time (qRT)-PCR analysis. n = 4 for wild-type and 3 for TNFR1 KO mice. *indicates p < 0.05. All bars indicate the mean ± SEM. **(D)** The number of macrophages, F4/80-positive cells, in IA walls of wild-type and TNFR1 KO mice. All bars indicate the mean ± SEM. The number of mice used is shown in parentheses. *indicates p = 0.0061.

First, we examined the activation of NF-kappaB in IA lesions by immunohistochemistry to detect the phosphorylated form of the NF-kappaB p65 subunit (Serine 536 residue), and confirmed the suppression of NF-kappaB activation under TNFR1 deficiency (Figure 3A). Next, we confirmed the suppression of MCP-1 and COX-2, which is a target gene of NF-kappaB signaling and also the crucial factor for IA formation [19,21], under TNFR1 deficiency, by both immunohistochemistry and quantitative real time (qRT)-PCR, and the differences between the two groups were statistically significant (immunohistochemistry: COX-2, wild-type compared to TNFR1-deficient mice, p = 0.021, n = 4 in each group; MCP-1, wild-type compared to TNFR1-deficient mice, p = 0.043, n = 4 in each group; qRT-PCR: COX-2, wild-type compared to TNFR1-deficient mice, p = 0.034, n = 4 in wild-type and 3 in TNFR1-deficient mice; MCP-1, wild-type compared to TNFR1-deficient mice, p = 0.034, n = 4 in wild type and 3 in TNFR1-deficient mice) (Figure 3B, C). Consistent with the suppression of MCP-1 in IA lesions from TNFR1-deficient mice (Figure 3B), the infiltration of macrophages into IA lesions was significantly inhibited in TNFR1-deficient mice (wild-type mice, 5.00 ± 0.31 cells, n = 7; TNFR1-deficient mice, 1.40 ± 0.68 cells, n = 5; p = 0.0061) (Figure 3D).

Taken together, these results suggest a crucial role for TNF-alpha-TNFR1 signaling in IA formation, by regulating inflammatory responses through the induction of NF-kappaB activation and subsequent macrophage infiltration into intracranial arteries.

## Discussion

In this study, we first revealed increased TNF-alpha content in IA lesions during IA formation and progression. Second, we clarified a crucial role for TNF-alpha-TNFR1 signaling in IA formation by regulating inflammatory responses via NF-kappaB activation, using TNFR1-deficient mice. Based on these experimental findings, we propose TNF-alpha-TNFR1 signaling as a potential therapeutic target for IAs.

A degenerative change in the tunica media is the histological feature of IA walls. Because the degenerative change in the tunica media in IA walls decreases arterial stiffness and, as a natural consequence, raises the probability of rupture, the mechanisms regulating the degeneration of media must be understood. In a previous study, the authors revealed the contribution of TNF-alpha to the phenotypic modulation of vascular smooth muscle cells, a major cell component of media [23]. They also demonstrated that TNF-alpha signaling shifted the phenotype of cultured vascular smooth muscle cells from contractile to synthetic, and induced the expression of pro-inflammatory factors, MCP-1, MMP-9, and IL-1beta [23]. The involvement of TNF-alpha signaling in phenotypic modulation and vascular wall alteration in vivo, after increased hemodynamic stress, was also clarified [23]. Combining these findings with the results presented here, it appears that the TNF-alpha-TNFR1 signaling cascade is a suitable therapeutic target for IAs in order to prevent rupture.

In this study, as well as previous studies using human specimens [6,12-14,23], the contribution of the TNF-alpha-TNFR1 signaling cascade to IA formation, progression, and possibly rupture, is revealed. Based on these findings, blocking the TNF-alpha-TNFR1 signaling cascade has great potential as a strategy for treating IAs. The TNF-alpha-TNFR1 signaling cascade is involved in the pathogenesis of other inflammatory diseases, such as rheumatoid arthritis. In rheumatoid arthritis, the inhibition of TNF-alpha-TNFR1 signaling by anti-TNF-alpha antibody or soluble TNF receptor has been established as an effective treatment for patients [24]. Given the effectiveness of TNF-alpha blockers in inflammatory settings, these drugs are good candidates for the treatment of IAs. However, as these drugs are expensive, and IAs have a high incidence in the general public and require a long treatment period, cheaper alternative drugs should be developed to inhibit TNF-alpha-TNFR1 signaling.

## Conclusions

In this study, we used rodent models of IA through increased hemodynamic stress to investigate the contribution of TNF-alpha-TNFR1 signaling to the pathogenesis of IA formation and progression. We confirmed an increase in TNF-alpha content in IA lesions in rat models during IA formation. Furthermore, TNFR1 deficiency suppressed IA formation by inhibiting inflammatory responses in IA walls such as NF-kappaB activation, MCP-1 induction, and macrophage infiltration. These results suggest a crucial role for TNF-alpha-TNFR1 signaling in IA formation and progression.

## Abbreviations

ACA: Anterior cerebral artery; IA: Intracranial aneurysm; MMP: Matrix metalloproteinase; OA: Olfactory artery; TACE: Tumor necrosis factor alpha converting enzyme; TNF-alpha: Tumor necrosis factor alpha; TNFR1: Tumor necrosis factor receptor superfamily, member 1a.

## Competing interests

The authors declare that they have no competing interests.

## Authors’ contributions

TA conceived the study, carried out the molecular biological experiments, secured grants, and drafted the manuscript. MF carried out the molecular biological experiments. MN and KN participated in the design and coordination of the study. SN conceived the study, secured grants, and participated in the design and coordination of the study. SN also helped to draft the manuscript. All authors read and approved the final manuscript.
